# The effect of dexmedetomidine on cerebral perfusion and oxygenation in healthy piglets with normal and lowered blood pressure anaesthetized with propofol-remifentanil total intravenous anaesthesia

**DOI:** 10.1186/s13028-017-0293-0

**Published:** 2017-05-03

**Authors:** Mai Louise Grandsgaard Mikkelsen, Rikard Ambrus, Rune Rasmussen, James Edward Miles, Helle Harding Poulsen, Finn Borgbjerg Moltke, Thomas Eriksen

**Affiliations:** 1 Department of Veterinary Clinical Sciences, Faculty of Health and Medical Sciences, University of Copenhagen, 16 Dyrlægevej, 1870 Frederiksberg C, Denmark; 2 Department of Surgical Gastroenterology C, Rigshospitalet, Faculty of Health and Medical Sciences, University of Copenhagen, 9 Blegdamsvej, 2100 Copenhagen Ø, Denmark; 3 Department of Neurosurgery, The Neuroscience Centre, Rigshospitalet, Faculty of Health and Medical Sciences, University of Copenhagen, 9 Blegdamsvej, 2100 Copenhagen Ø, Denmark; 4 Department of Neuroanaesthesia, Rigshospitalet, University of Copenhagen, 9 Blegdamsvej, 2100 Copenhagen Ø, Denmark; 5 Department of Anaesthesia, Bispebjerg and Frederiksberg Hospitals, Faculty of Health and Medical Sciences, University of Copenhagen, 23 Bispebjerg Bakke, 2400 Copenhagen NV, Denmark

**Keywords:** Cerebral perfusion, Cerebral oxygenation, Swine, Propofol, Remifentanil, Dexmedetomidine, Laser speckle contrast imaging, Licox

## Abstract

**Background:**

During anaesthesia and surgery, in particular neurosurgery, preservation of cerebral perfusion and oxygenation (CPO) is essential for normal postoperative brain function. The isolated effects on CPO of either individual anaesthetic drugs or entire anaesthetic protocols are of importance in both clinical and research settings. Total intravenous anaesthesia (TIVA) with propofol and remifentanil is widely used in human neuroanaesthesia. In addition, dexmedetomidine is receiving increasing attention as an anaesthetic adjuvant in neurosurgical, intensive care, and paediatric patients. Despite the extensive use of pigs as animal models in neuroscience and the increasing use of both propofol-remifentanil and dexmedetomidine, very little is known about their combined effect on CPO in pigs with uninjured brains. This study investigates the effect of dexmedetomidine on CPO in piglets with normal and lowered blood pressure during background anaesthesia with propofol-remifentanil TIVA. Sixteen healthy female Danish pigs (crossbreeds of Danish Landrace, Yorkshire and Duroc, 25–34 kg) were used. Three animals were subsequently excluded. The animals were randomly allocated into one of two groups with either normal blood pressure (NBP, n = 6) or with induced low blood pressure (LBP, n = 7). Both groups were subjected to the same experimental protocol. Intravenous propofol induction was performed without premedication. Anaesthesia was maintained with propofol-remifentanil TIVA, and later supplemented with continuous infusion of dexmedetomidine. Assessments of cerebral perfusion obtained by laser speckle contrast imaging (LSCI) were related to cerebral oxygenation measures (P_br_O_2_) obtained by an intracerebral Clark-type Licox probe.

**Results:**

Addition of dexmedetomidine resulted in a 32% reduction in median P_br_O_2_ values for the LBP group (P = 0.03), but no significant changes in P_br_O_2_ were observed for the NBP group. No significant changes in LSCI readings were observed in either group between any time points, despite a 28% decrease in the LBP group following dexmedetomidine administration. Caval block resulted in a significant (P = 0.02) reduction in median MAP from 68 mmHg (range 63–85) at PCB to 58 mmHg (range 53–63) in the LBP group, but no significant differences in either P_br_O_2_ or LSCI were observed due to this intervention (P = 0.6 and P = 0.3 respectively).

**Conclusions:**

Addition of dexmedetomidine to propofol-remifentanil TIVA resulted in a significant decrease in cerebral oxygenation (P_br_O_2_) measurements in piglets with lowered blood pressure. Cerebral perfusion (LSCI) did not decrease significantly in this group. In piglets with normal blood pressure, no significant changes in cerebral perfusion or oxygenation were seen in response to addition of dexmedetomidine to the background anaesthesia.

**Electronic supplementary material:**

The online version of this article (doi:10.1186/s13028-017-0293-0) contains supplementary material, which is available to authorized users.

## Background

During anaesthesia and surgery, in particular neurosurgery, preservation of cerebral perfusion and oxygenation (CPO) is essential for normal postoperative brain function [1, 2]. Choice of anaesthetic protocol, cerebral pathology, the patient’s haemodynamic stability, and their interaction can influence the CPO response [3, 4]. Studies in rabbits have shown significant differences in the cerebral perfusion responses to anaesthetics in normal and injured brains [5, 6], so CPO results from neurocritical studies cannot be definitively translated to patients with normal brain physiology. In humans over 60 years, both short and long-term postoperative cognitive disorders can be seen following anaesthesia in relation to both non-cardiac and non-neurologic major surgery [7]. In this context, understanding of the anaesthetic effect on CPO in the healthy brain must be considered a prerequisite for understanding the pathology and anaesthetic influence in the diseased brain [3]. Total intravenous anaesthesia (TIVA) with a combination of propofol and remifentanil is extensively used during ambulatory surgery, paediatric surgery and neurosurgery in humans [8–11]. This combination of anaesthetics has several characteristics that preserve cerebrovascular reactivity to CO_2_ and autoregulatory mechanisms [11–14], and can be a suitable basis anaestesia in experimental CPO studies.

In this context, alpha_2_-adrenergic-agonists, in particular dexmedetomidine, are receiving increasing attention as sedatives and as anaesthetic adjuvants for neurosurgical, intensive care and paediatric human patients [15–19]. In veterinary anaesthesia, α_2_-adrenergic-agonists have been used widely for sedation and as part of general anaesthesia for many years [20–22]. Dexmedetomidine potentiates propofol and opioids and has been shown to reduce cerebral blood flow (CBF), possess neuroprotective properties, and to be suitable for continuous infusion in TIVA and conscious sedation in humans [17, 23–26]. In spite of this, the use of dexmedetomidine in patients with cardiovascular disease or cerebral pathology is still debated primarily with regards to systemic hypotension, bradycardia and potential cerebral vasoconstriction [27].

Both in human and veterinary anaesthesia it is recommended that these patients need individually tailored neuroanaesthesia in order to maintain optimal CPO [21, 28, 29]. Due to ethical concerns regarding clinical studies in humans, strategies for individually tailored neuroanesthesia are often explored in experimental animals. Porcine animal models are well described in neuroscience and are often preferred over smaller animals due to the closer resemblance to the human brain in anatomy, growth, and development [30]. Despite the extensive use of pigs as animal models and the abundant use of propofol-remifentanil in human neuroanaesthesia and dexmedetomidine in veterinary anaesthesia in general, very little is known about their combined effect on CPO in anaesthetized pigs with uninjured brains. Guidelines for anaesthesia in experimental animal trials studying CPO are, therefore, poorly established [31].

The objective of this study was to investigate the effect of dexmedetomidine on CPO in piglets with normal and lowered blood pressure during background anaesthesia with propofol-remifentanil TIVA.

## Methods

### Study design

**Fig. 1:**
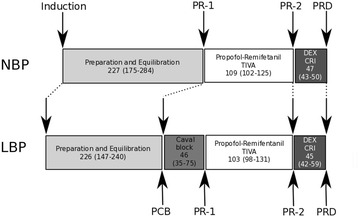
Experimental design. The experimental flow for the two groups (*NBP* normal blood pressure group, *LBP* low blood pressure group), with the median duration of each period (min–max range in brackets). Key time points are marked (*arrows*), and corresponding time points between groups are joined by *dotted lines*. *PCB* Pre-caval Block, *PR-1* Propofol-remifentanil experimental start, *PR-2* Propofol-remifentanil end–immediately prior to dexmedetomidine infusion, *PR-D* following dexmedetomidine infusion

This study was designed as a non-blinded, two-arm parallel group, experimental animal trial. Animals were allocated into one of two groups with either normal blood pressure (NBP) or with low blood pressure (LBP) by a computerized lottery draw randomization schedule (www.random.org ). Figure 1 illustrates the experimental protocol; additional data will be reported separately. Full details and data regarding the entire study are presented in Additional file 1. The Danish Animal Experiments Inspectorate approved the study (licence no. 2013-15-2934-00909), and all procedures were performed in accordance with national legislation and The Council of Europe Convention ETS 123. All animals were acquired through, and housed by, the Department of Animal Experimental Medicine at the University of Copenhagen. The piglets were inspected and considered healthy on reception at the housing facility by the veterinarian responsible for the trial. The piglets were allowed to acclimatize to the facility for at least 1 week before the experiment began. The piglets were housed in groups of 3–4 (in the same group as they arrived) in cement-floored pens measuring 5.5 m^2^ with fine wood chip and straw bedding. Environmental enrichment with toys and music were supplied through the daytime. A 7:17 light:dark cycle was used and the room temperature were kept at 19–20 °C. The piglets were fed a commercial finisher diet (Svinefoder 5, Nordsjællands Andels Grovvareforening Amba, Helsinge, Denmark), given according to weight twice daily (0800 and 1500), and the piglets had free access to water. Food, but not water, was withheld overnight prior to the experiment.

The results of this study are reported in accordance with ARRIVE guidelines [32].

### Animals and anaesthesia

Sixteen healthy female Danish pigs (crossbreeds of Danish Landrace, Yorkshire and Duroc) with a median weight of 27 kg (range 25–34) were used. Venous access for propofol induction was secured the day prior to the experiment under sedation (see Additional file 2), using catheters (Arrow^®^ arterial catheterization set, SAC-00822: 20 ga × 8 cm, Teleflex, Ireland) placed in the auricular vein of both ears.

All 16 animals were subjected to the same anaesthetic protocol: no premedication was given on the day of the experiment. General anaesthesia was induced by an intravenous (iv) bolus of 4–8 mg/kg propofol (10 mg/ml Propofol “B. Braun”, B. Braun Melsungen, Germany), while the animals were still in their pen to minimise stress. After transport to the experimental area, they were placed in ventral recumbency on a padded operating table and mechanically ventilated following endotracheal intubation. A mechanical ventilator (Bag-in-bottle respirator, Type no. 86,298, Dameca A/S, Roedovre, Denmark) was used to maintain end tidal CO_2_ (EtCO_2_) between 35 and 50 mmHg by regulation of the respiration rate (RR) (11–21/min) and tidal volume (10–15 ml/kg) to keep the inflation pressure below 20 cm H_2_O using an inspiration:expiration ratio of 1:2 and a set PEAK pressure of 20 cm H_2_O. The circuit used was a co-axial circle-system.

General anaesthesia was maintained by propofol (10 mg/ml Propofol “B. Braun”, B. Braun Melsungen, Germany) and remifentanil (1 mg/ml Ultiva^®^, GlaxoSmithKline Pharma, Denmark, diluted with sterile water to 100 µg/ml) TIVA using separate syringe pumps (Terumo, Terufusion Syringe Pump TE-331, Belgium). Doses were based on our pilot studies, with a propofol dose range of 12–20 mg/kg/h and a remifentanil dose range of 20–45 µg/kg/h. Doses were individually regulated to accomplish unresponsiveness to noxious stimuli (dewclaw pinching). Propofol doses were adjusted to control anaesthetic depth (assessed as lack of movement) and remifentanil doses were adjusted to eliminate responses to noxious stimuli. Propofol and remifentanil doses were not altered after animal preparation was completed. Dexmedetomidine (0.5 mg/ml Dexdomitor^®^, Orion Pharma Animal Health, Sollentuna, Sweden) was added following time point PR2 (Fig. 1) with an iv bolus of 1 µg/kg administrated over 10 min, followed by a fixed dose of 0.7 µg/kg/h iv (5 µg/ml solution).

### Surgical preparation and instrumentation

All invasive procedures were conducted after local infiltration with a mixture of lidocaine (20 mg/ml Xylocain^®^ AstraZeneca, Copenhagen, Denmark) and bupivacaine (5 mg/ml Marcain^®^ AstraZeneca, Copenhagen, Denmark).

A circular craniotomy (20–30 mm) was performed over the right parietal lobe with a 5 mm craniotome (Midas Rex, Medtronic, Heerlen, The Netherlands). After dural excision, cerebral perfusion was directly measured semi-quantitatively (laser speckle perfusion units—LSPU) using a laser speckle contrast imaging (LSCI) camera (moorFLPI-2, Moor Instruments, Devon, UK) placed perpendicular to and 25 cm from the region of interest. The position of the head of the animal remained unchanged throughout the experiment. An intracerebral Clark-type probe connected to a Licox^®^ Brain Tissue Oxygen Monitoring System (Integra LifeSciences, New Jersey, USA) was placed in the periphery of the craniotomy, in white matter 25 mm subdurally and secured to the craniotomy edge with bone wax for recording of cerebral oxygenation (partial pressure of oxygen in brain tissue—P_br_O_2_).

The femoral artery was cannulated (Arrow^®^ arterial catheterization set, SAC-01620: 20 ga × 16 cm, Teleflex, Ireland) for invasive blood pressure monitoring and intermittent blood collection for blood gas analysis (GEM Premier 3000, Instrumentation Laboratory, Lexington MA, USA). The femoral vein was cannulated for placement of an 8 French balloon-tipped catheter (MILA International Inc., Kentucky, USA) in all animals but only used for induction of caval block in the LBP group. The catheter was premeasured to position the balloon in the caudal vena cava just caudal to the heart. Caval block was induced by injecting sterile isotonic NaCl into the catheter balloon to partially obstruct the vena cava.

A urinary catheter with a closed collecting bag was placed to prevent bladder distension. Fluids were administered throughout the experiment at 2.5 ml/kg/h iv (glucose 50 mg/ml, B. Braun Melsungen. Germany).

A multiparametric bedside monitor (Datex-Ohmeda S/5, Helsinki, Finland) was used to record cardiovascular and pulmonary variables every 30 s: data were transferred to a personal computer using Datex-Ohmeda S/5™ Collect software (GE Healthcare, Helsinki, Finland). Recorded variables were pulse rate, invasive mean arterial blood pressure (MAP), body temperature by oesophageal probe, fraction of inspired oxygen (FiO_2_) and EtCO_2_. The electrocardiogram and peripheral oxygen saturation by pulse oximetry (SpO_2_) measured on the tail or the lower lip were monitored for continuous assessment.

### Experimental protocol

After instrumentation the Licox probe was equilibrated for up to 2 h or until P_br_O_2_ exceeded 25 mmHg before baseline data were collected (PR-1–NBP and PCB–LBP) for all animals (Fig. 1). In the LBP group, MAP was then lowered by caval block until a stable MAP of 50–60 mmHg was reached, and data recording was repeated (PR1–LBP). The caval block was maintained unadjusted throughout the experiment. Data were collected at all key time points (Fig. 1), with time point PRD placed following 30 min of dexmedetomidine infusion. Arterial blood gas samples for analysis of pH, P_a_CO_2_, P_a_O_2_, bicarbonate (HCO_3_^−^), base excess (BE), blood glucose, lactate, total haemoglobin concentration (THbc) and haematocrit (Hct) were obtained at each time point.

Between PR-1 and PR-2, and following PRD, vasopressor challenges were made using norepinephrine and phenylephrine. A 30-min washout period was allowed before time point PR2. Data collected during these periods of vasopressor challenge will be reported separately.

At the end of the experiments, the animals were euthanized with pentobarbital sodium iv (400 mg/ml, Euthasol vet., Virbac, Denmark).

### Statistics

Statistical per protocol analysis was performed using SPSS 24.0 software (IBM® SPSS® Statistics for Mac, IBM Corp. ©, Armonk, NY, USA), and Microsoft^®^ Excel^®^ for Mac 2011 version 14.3.9 (2010 Microsoft Corporation). Normal distribution of data could not be assumed due to the sample size and non-parametric statistical analyses were used. Data are reported as median values with either range (min–max) or 95% confidence intervals (95% CI) obtained using Hodges–Lehmann estimates given where appropriate. P-values ≤0.05 were considered statistically significant. Comparisons between time-points PCB and PR-1 (LBP group) were made using Wilcoxon’s signed rank test. Comparisons between LBP and NBP were made using the independent-samples median test. Comparisons of primary outcome measures (P_br_O_2_ and LSPU) and secondary explanatory variables (haemodynamics and blood gas) across time points PR-1 to PR-2 and PR-2 to PRD were made using Friedman’s ANOVA with post hoc Bonferroni corrected pairwise comparisons when Friedman’s ANOVA was significant. Comparisons between selected time point medians and literature-derived cut-off values were made using the one-sided, one-sample Wilcoxon signed rank test to determine if medians lay above the cut-off. A sample size of 16 animals, divided into two groups of 8, was calculated using conservative estimates based on earlier studies [33] with expected power of 80% in detecting a minimum of 30% difference in MAP with a two-tailed significance level of 5% after supplementation of dexmedetomidine.

## Results

All 16 animals completed the experimental protocol. Data from three (NBP group n = 2, LBP group n = 1) piglets were excluded from analysis. One piglet developed signs of brain oedema with a severe reduction in CPO following craniotomy (LBP group). In the NBP group, one pig had persistently and unexplainably high pulse rate, EtCO_2_, P_a_CO_2_ and a low pH, which were expected to produce an atypical CPO response. The other pig was excluded due to technical difficulties.

Anaesthesia time, preparation time, anaesthetic doses and baseline CPO measurements were not statistically different between NBP and LBP groups. Both groups reached normal cerebral oxygenation levels following animal preparation and equilibration of the Licox probe (Table 1) [8, 34].

**Table 1:** Baseline data. Data were recorded at time points PR1 (normal blood pressure group—NBP) and PCB (low blood pressure group—LBP), prior to induction of caval block in the LBP group

	Propofol-remifentanil 1 (PR-1)	Pre-caval block (PCB)	P value
	NBP (n = 6)	LBP (n = 7)	
Preparation time (min)	227 (175–284)	226 (147–240)	0.63
Propofol dose (mg/kg/h)	15 (12–20)	15 (12–18)	1.00
Remifentanil dose (µg/kg/h)	30 (20–40)	25 (20–45)	0.53
Cerebral oxygenation (P_br_O_2_ mmHg)	25.6 (19.9–55.7)	29.6 (20.3–32.2)	0.45
Cerebral perfusion (LSPU)	1033.9 (730.4–1251.5)	1090.7 (845.5–1762.0)	0.53

Data are reported as median and range (min–max)

P_br_O_2_, partial pressure of oxygen in brain tissue; LSPU, laser speckle perfusion unit

**Fig. 2:**
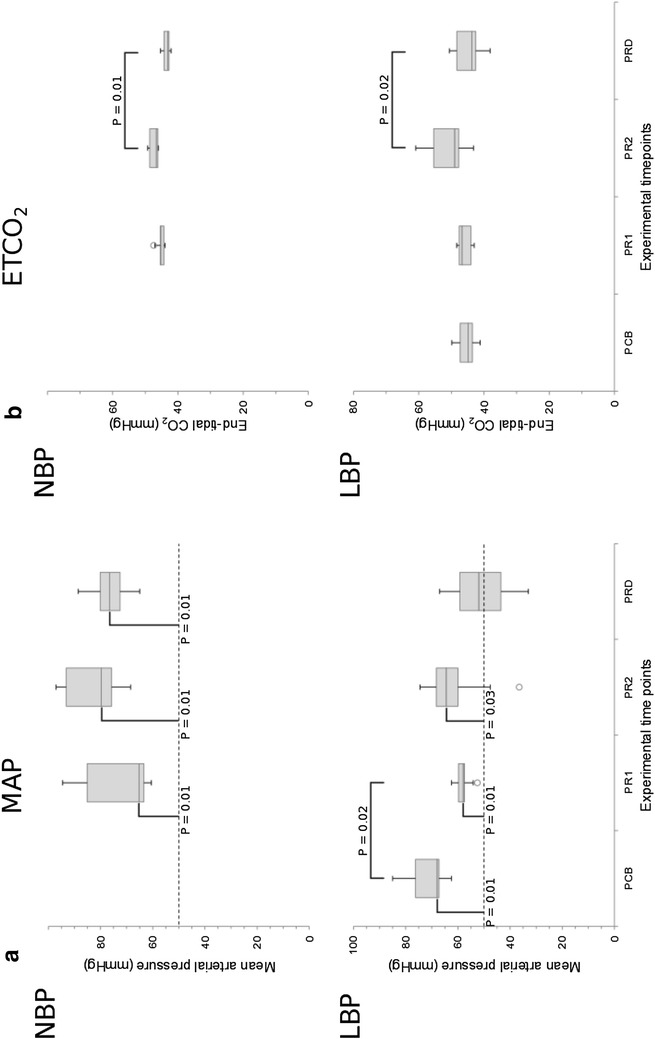
*Boxplots* of mean arterial blood pressure and end-tidal carbon dioxide at the different timepoints. *Box* and *whisker* plots showing median (line), interquartile range (*shaded*) and outliers (*circles*) for mean arterial pressure (in mmHg) (**a**), and end-tidal carbon dioxide (ETCO_2_, in mmHg) (**b**) in the normal blood pressure group (NBP) and low blood pressure group (LBP).* Whiskers* extend a maximum of 1.5× the interquartile range. The lower mean arterial pressure limit for cerebral autoregulation is shown (*dashed line*). Experimental timepoints: *PCB* pre-caval block, *PR-1* initial baseline for propofol-remifentanil TIVA, *PR-2* immediately before dexmedetomidine administration, *PRD* following propofol-remifentanil-dexmedetomidine TIVA. Significant differences from either the autoregulation limit (*vertical bars*) or between timepoints (*horizontal bars*) are shown

**Fig. 3:**
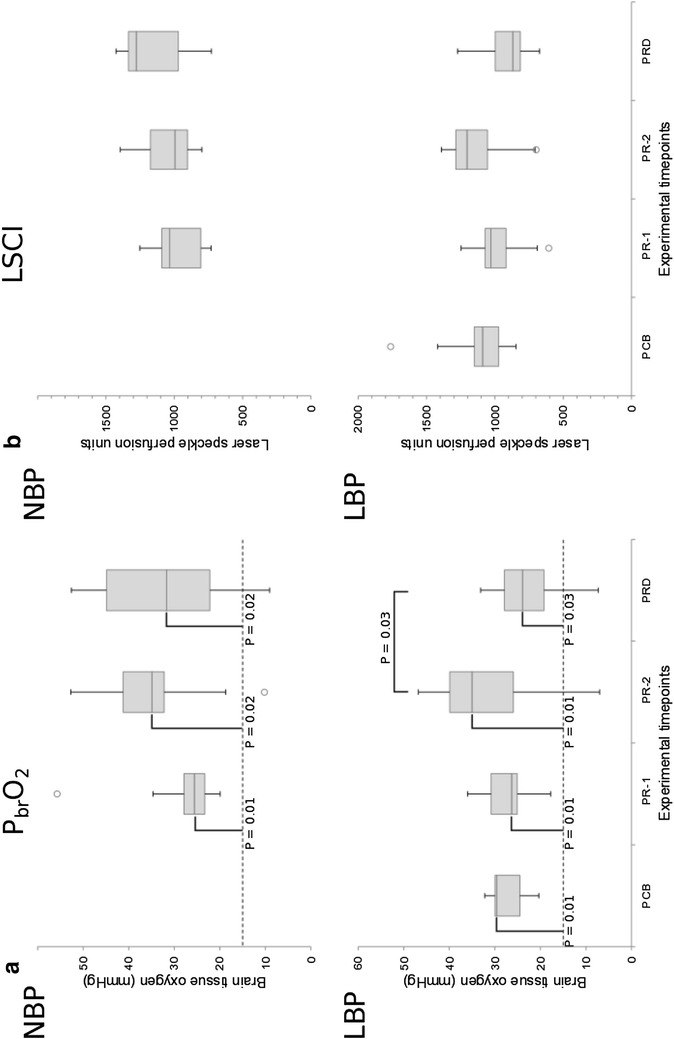
*Boxplots* of cerebral perfusion and oxygenation at the different time points. *Box* and *whisker plots* showing median (*line*), interquartile range (*shaded*) and outliers (*circles*) for partial pressure of tissue oxygen (P_br_O_2_, in mmHg) (**a**) and laser speckle perfusion units (**b**) in the normal blood pressure group (NBP) and low blood pressure group (LBP).* Whiskers* extend a maximum of 1.5× the interquartile range. The value for ischaemic threshold is shown (*dashed line*). Experimental time points: *PCB* pre-caval block, *PR-1* initial baseline for propofol-remifentanil TIVA, *PR-2* immediately before dexmedetomidine administration, *PRD* following propofol–remifentanil–dexmedetomidine TIVA. Significant differences from either the ischaemia limit (*vertical bars*) or between timepoints (*horizontal bars*) are shown

Caval block resulted in a significant (P = 0.02) reduction in median MAP from 68 mmHg (range 63–85) at PCB to 58 mmHg (range 53–63) at PR1 in the LBP group (Fig. 2), but no significant differences in either P_br_O_2_ or LSPU were observed due to this intervention (P = 0.6 and P = 0.3 respectively) (Fig. 3).

A significant difference in the intra-group distributions of P_br_O_2_ readings was observed between time points PR-1, PR-2 and PRD for group LBP (P = 0.05), but not for NBP (P = 0.5). Post-hoc Bonferroni corrected pairwise comparisons showed a significant (P = 0.03) 32% reduction in median P_br_O_2_ values (−7 mmHg, 95% CI −2; −17) for LBP between PR-2 and PRD. Median P_br_O_2_ readings for both groups were significantly (P < 0.03, one-tailed) higher than the threshold value of 15 mmHg for cerebral ischaemia at all time points [35, 36]. No significant changes in the distributions of LSPU readings were observed for either group between time-points PR-1, PR-2 and PRD, despite a 28% fall in LSPU values (median difference −186 LSPU, 95% CI −7; −478) in the LBP group following dexmedetomidine administration. No significant between-group differences were detected at any time point.

In the LBP group, median MAP was significantly (P < 0.03, one-tailed) greater than the lower limit for cerebral autoregulation (LLCA) of 50 mmHg at all time points except PRD, but in the NBP group median MAP was consistently above this limit (P = 0.01, one-tailed).

No statistically significant intra-group changes in MAP over time points PR-1, PR-2 and PRD were observed, despite a trend to an increase from PR-1 to PR-2 (median 6 mmHg, 95% CI −10; 13 and 9 mmHg, 95% CI −4; 25 for LBP and NBP, respectively) and a decrease from PR-2 to PRD (median −10 mmHg, 95% CI −1; −22 and −6 mmHg, 95% CI −18; 5). This corresponds to a 19% reduction of median MAP in the LBP group following dexmedetomidine infusion. At all time points, median MAP values were significantly (P = 0.03) lower for the LBP group than for the NBP group, with median differences of 8−25 mmHg.

In the LBP group, there was a significant (P = 0.02) increase in median pulse rate following initiation of caval block (median increase 10, 95% CI 2; 47). No significant differences were found between time points PR1, PR2 and PRD for either group, or between groups at any time point.

For both LBP and NBP groups, a significant (P = 0.02 for both) intra-group change in EtCO_2_ distributions was observed. Pairwise testing showed a significant decrease in median values of 5 mmHg (95% CI 2; 11) for LBP (P = 0.02) and 4 mmHg (95% CI 2; 5) for NBP (P = 0.01) between time points PR-2 and PR-D. This was associated in the LBP group, but not the NBP group, with a significant (P = 0.03) decrease in median PaCO_2_ readings of 5 mmHg (95% CI 2.5; 9) from PR-2 to PR-D. A trend to increasing EtCO_2_ levels in the LBP group between PR-1 and PR-2 was associated with a significant (P = 0.03) increase in P_a_CO_2_ levels (median increase 6, 95% CI 1; 11). Median P_a_CO_2_ values were 0-3.5 mmHg higher, and median EtCO_2_ values 0.6–2 mmHg higher, in the LBP group than the NBP group, but this was not statistically significant at any time point.

Median body temperature was 0.8–1.5 °C higher in the LBP group than the NBP group across time points PR-1, PR-2 to PRD, but this was not statistically significant (Table 2). No significant intra-group changes in distribution were observed.

**Table 2:** Physiologic, anaesthetic and blood gas data for the two experimental groups (low blood pressure—LBP and normal blood pressure—NBP) at key study time points

Group	Pre-caval block (PCB)	Propofol-remifentanil 1 (PR-1)		Propofol-remifentanil 2 (PR-2)		Propofol-remifentanil-dexmedetomidine (PRD)		Reference ranges
	LBP	NBP	LBP	NBP	LBP	NBP	LBP	
Body temperature (°C)	38.6 (37.5–40.0)	37.9 (37.3–38.9)	38.7 (37.5–39.9)	38.3 (37.7–39.3)	39.7 (38.0–40.9)	38.3 (37.2–39.3)	40 (38.1–40.1)	38.7–39.8 [37]
FIO_2_ (%)	84 (82–87)	83 (78–85)	85 (82–87)	84 (80–85)	86 (83–87)	84 (79–85)	85 (83–87)	–
Pulse rate (beats/min)	80 (54–104)	67 (57–85)	87^a^ (58–137)	80 (68–85)	114 (67–179)	78 (51–111)	111 (71–153)	70–120 [37]
ETCO_2_ (mmHg)	45 (41–50)	45 (44–47)	47 (43–48)	47 (46–49)	49 (43–61)	43^a^ (42–45)	44^a^ (38–51)	35–50 mmHg [41]
pH^1^	7.4 (7.34–7.46)	7.43 (7.38–7.47)	7.41 (7.35–7.46)	7.41 (7.31–7.46)	7.32^b^ (7.25–7.46)	7.46^a^ (7.39–7.49)	7.37^a^ (7.29–7.48)	7.4–7.43[38];7.4–7.53 [39]
pCO_2_ (mmHg)^1^	49 (48–56)	51 (48–52)	50 (45–55)	53 (49–60)	56^a^ (45–68)	51 (48–52)	52^a^ (44–55)	41.8–49.1 [38]
pO_2_ (mmHg)^1^	443 (432–482)	449 (431–507)	463 (411–487)	452 (433–485)	447 (426–498)	455 (422–497)	480 (434–498)	18.8–39.1 [38]
HCO_3_^−^ (mmol/l)	31 (28–34)	33 (30–35)	30 (28–34)	33 (30–37)	29 (25–32)	35 (31–38)	30^b^ (24–33)	24.9–30.3 [38] 22–33 [39]
Base excess (mmol/l)	6 (3–11)	9 (5–12)	5 (3–10)	10 (4–14)	4 (−1– 9)	11 (7–15)	5^b^ (−2–10)	1.3–5.1(+) [38]
Glucose (mmol/l)	4.9 (3.9–5.6)	5.4 (4.8–6.9)	5.5^a^ (4.8–8.0)	7.2 (4.9–8.4)	9.6 (4.4–14.7)	5.2 (3.7–5.9)	6.4 (4.3–9.5)	2.6–6.5 [39]
Lactate (mmol/l)	0.5 (0.4–2.1)	0.9 (0.7–1.0)	0.9^a^ (0.4–2.4)	0.7 (0.3–1.4)	1.2 (0.7–3.0)	0.5 (0.3–0.9)	1.3 (0.5–3.9)	0.5–1.5 [39]; 0.5–5.5 [40]
Total haemoglobin (mmol/l)	4.6 (4.6–5.6)	4.6 (4.6–6.1)	5.0^a^ (4.6–6.1)	4.9 (4.6–6.1)	5.6^a^ (5.0–7.0)	4.7 (4.2–6.1)	5.2 (4.6–6.0)	6.21–9.93 [37]
Haematocrit (%)	24 (24–29)	24 (24–32)	26^a^ (24–32)	26 (24–32)	29^a^ (26–36)	25 (22–32)	27 (24–31)	29.5–45.9 [42]

Median values are shown with range (min–max)

For the LBP group, statistically significant differences were pulse rate (P = 0.02, median increase 10, 95% CI 2; 47), ETCO_2_ (P = 0.02, median decrease 5 mmHg, 95% CI 2; 11), pH (P = 0.05, median increase 0.04, 95% CI 0.01; 0.08), pCO_2_ (P = 0.03, median increase 6 mmHg, 95% CI 1; 11, and P = 0.03, median decrease 5 mmHg, 95% CI 3; 9), glucose (P = 0.05, median increase 1.2 mmol/l, 95% CI 0.0; 2.7, and P = 0.02, median increase 3.5 mmol/l, 95% CI 0.8; 7.1), lactate (P = 0.04, median increase 0.2 mmol/l, 95% CI 0.0; 1.0), total haemoglobin (P = 0.05, median increase 0.4 mmol/l, 95% CI 0.0; 0.8 and P = 0.02, median increase 0.5, 95% CI 0.2; 1.2), and haematocrit (P = 0.05, median increase 2%, 95% CI 0; 4, and P = 0.02 (median increase 3%, 95% CI 1; 6). For the NBP group, statistically significant differences were ETCO_2_ (P = 0.01, median decrease 4 mmHg, 95% CI 2; 5) and pH (P = 0.01, median increase 0.05, 95% CI 0.02; 0.08). Significant intergroup differences were pH (P = 0.03, median difference 0.09, 95% CI 0.01; 0.15), HCO_3_^−^ P = 0.03 (median difference 6 mmol/l, 95% CI 2; 9) and base excess (P = 0.03, median difference 7 mmol/l, 95% CI 2; 12)

^1^Temperature corrected values

^a^Statistically significant differences from the previous study time point

^b^Statistically significant differences from the other experimental group within the same time point

Additional physiological parameters are reported in Table 2.

## Discussion

The key finding in this study was the significant fall in cerebral oxygenation following supplementation with dexmedetomidine to propofol-remifentanil TIVA in piglets with lowered blood pressure but not in piglets with normal blood pressure.

### Cerebral perfusion and oxygenation response to dexmedetomidine

In the piglets with normal blood pressure neither cerebral perfusion nor oxygenation changed significantly after addition of dexmedetomidine to propofol-remifentanil TIVA. This supports previous findings that dexmedetomidine does not affect P_br_O_2_ in patients with normal blood pressure and healthy brains [19] or patients with neurovascular injuries [43].

Cerebral oxygenation measured by Licox decreased after dexmedetomidine supplementation in piglets with lowered blood pressure. The normal range for P_br_O_2_ is 25–30 mmHg in pigs [34], which is comparable to the human normal range of 25–50 mmHg [8]. Our median baseline results were within these limits. Caval block in the LBP group produced a significant decrease in MAP, but not in the CPO measures. The lack of CPO response to induced hypotension was expected, since the caval block was adjusted to a MAP target range of 50–60 mmHg in order to keep the blood pressure within the standard range of cerebral autoregulation [12]. The subsequent significant 32% decrease in cerebral oxygenation in the LBP group is most likely a response to the additive effect of dexmedetomidine to propofol-remifentanil TIVA.

Cerebral oxygenation measured as partial pressure of oxygen in brain tissue (P_br_O_2_) is believed to reflect factors affecting both oxygen cerebral tissue diffusion as well as CBF [35] and may thus be regarded as an indicator of the combined effect of cerebral perfusion and metabolism [44]. Dexmedetomidine has been shown to decrease CBF in both human [25, 26, 45, 46] and animal studies [47, 48] but did not reduce P_br_O_2_ in a series of five surgical patients with neurovascular injuries [43]. The same studies conclude that the reduction in CBF is not coupled to a decrease in cerebral metabolic rate. The influence of dexmedetomidine on CBF is believed to be due to lowering of systemic blood pressure through centrally α_2A_-mediated sympatholysis [49, 50] or by α_2B_-mediated cerebral vascular smooth muscle constriction [26, 51] as well as lowering cerebral metabolism by lowering brain activity [26]. The overall clinical effect reflects the sum of these central and peripheral effects, and is related to the doses used [52, 53].

In this study, cerebral perfusion was evaluated by LSCI. Laser speckle contrast imaging is able to assess real-time cerebral perfusion [54–56], and in piglets LSCI has been successfully used to evaluate changes in pial arteriolar blood flow [57]. Our results disagree with previous studies [25, 26, 45–48], since addition of dexmedetomidine did not produce significant changes in cerebral perfusion in either the NBP or the LBP group. The significant decrease found in P_br_O_2_ in the latter group, could not be related to a statistically significant decrease in LSPU measures, despite a decrease in 6 out of 7 piglets resulting in a 30% reduction of median LSPU. A post hoc power analysis (G*power for Mac, version 3.19.2) based on our LSPU data revealed a low power (0.47), increasing the risk of Type II error, so that an effect on cerebral perfusion by dexmedetomidine might have been falsely rejected. Since we did not assess cerebral metabolism, we cannot conclude if the significant P_br_O_2_ reduction was related to reduced CBF or to a lack of reduction in cerebral metabolism.

All P_br_O_2_ measurements were significantly higher than 15 mmHg, which may be used as a threshold value below which the risks of cerebral ischaemia [35], and mortality may increase [36].

The clinical significance of reduced cerebral oxygenation will probably depend on the P_br_O_2_ prior to dexmedetomidine supplementation and to the integrity of cerebral autoregulatory mechanisms, as well as to haemodynamic stability. Therefore, particular attention should be directed towards paediatric, neurosurgical, and intensive care patients, which are patient groups in which the use of dexmedetomidine has gained increasing interest in recent years [15, 17, 45, 58, 59].

### Haemodynamic responses

The baseline MAP was comparable to findings in other studies based on propofol-remifentanil in pigs [60], even though it was relatively low. The significant difference in MAP between the NBP and the LBP group was consequently clinically small, but the difference was consistent and statistically significant throughout the experiment.

The MAP in the NBP group remained well within normal limits and statistically significantly exceeded the LLCA at all time points, indicating that in piglets with normal blood pressure propofol-remifentanil TIVA both with and without dexmedetomidine provides a haemodynamically stable anaesthesia (Fig. 2). In contrast, in the LBP group following addition of dexmedetomidine the median MAP no longer significantly exceeded the LLCA. Although studies on propofol-remifentanil-dexmedetomidine anaesthesia in pigs are not available, Sano et al. [33] showed a significant decrease in MAP with addition of the same dose of dexmedetomidine to background anaesthesia of propofol alone, in contrast to our findings in the NBP group. The observed 19% decrease in MAP after addition of dexmedetomidine in the LBP group, even though not statistically significant, corresponds to previous reports in humans [50, 61, 62] and is mediated by activation of central α_2_-adrenergic receptors and subsequent attenuation of sympathetic activity [63]. Thus, individuals with compromised cardiovascular function may be at risk of experiencing an additional decrease in MAP when dexmedetomidine is added to propofol-remifentanil TIVA, which may be a concern in both experimental and clinical settings.

The significant increase in pulse rate between PCB and PR-1 observed in the LBP group likely represents an intact sympathetic response during propofol-remifentanil TIVA. The lack of pulse increase in response to the apparent decrease in MAP between PR-2 and PRD could reflect the sympatholytic effect of dexmedetomidine.

### Background physiology

Mild to moderate hypercapnia was observed in both NBP and LBP groups, with minimal and statistically insignificant inter-group differences. Previous studies suggest that even small increases in P_a_CO_2_ can increase cerebral blood flow [26]. Mild compensated respiratory acidosis was observed in both groups until PR-1. However, the slightly higher P_a_CO_2_ levels in the LBP group and the mild acidosis was more evident in the LBP group compared to the NBP group at time point PR-2, but this did not appear to have any significant consequences for cerebral perfusion in our study.

Throughout the study, both THbc and haematocrit were below the reference range for adult pigs, but this is expected in piglets of 2–3 months of age [64]. In the LBP group both parameters increased significantly from PCB to PR1 and PR1 to PR2, but the amounts were small and not considered clinically relevant to this study.

### Strengths and limitations

The model, experimental design and sample size in this study, were selected to investigate the effects of both dexmedetomidine and hypotension and to follow the 3 Rs tenet for the use of animals in research in the best possible way [65]. Some power was lost due to the post hoc exclusion of three animals.

The porcine model presented here was technically feasible, effectively inducing hypotension, which persisted throughout an experimental time period exceeding 7 h. The craniotomy was technically simple, but required careful preparation and the only complication encountered was a small haematoma in one animal. This complication represents the main methodological concern about LSCI recording in this model.

Avoiding premedication on the experimental day eliminates interference from other drugs on the effects of propofol, remifentanil and dexmedetomidine. Additional sedative or anaesthetic drugs risk contaminating cerebral outcome measures in animal experiments unless proper consideration is given to sufficient washout periods and drug interactions [8, 66–68].

In terms of neural maturity, this animal model best mirrors cerebral haemodynamics in children of approximately 10 months of age [69, 70] and translation to other age groups should be made with caution [71, 72].

We cannot exclude a potential influence from the vasopressor challenges performed on these animals, since we have not included a vasopressor-free control group. However, we used an extended washout period after vasopressor treatment (see Additional file 1), and no significant differences were noted for the primary outcomes measures of CPO between PR-1 and PR-2.

## Conclusions

Addition of dexmedetomidine to propofol-remifentanil TIVA resulted in a significant decrease in cerebral oxygenation (P_br_O_2_) measurements in piglets with lowered blood pressure. Cerebral perfusion (LSCI) did not decrease significantly in this group. In piglets with normal blood pressure, no significant changes in cerebral perfusion or oxygenation were seen in response to addition of dexmedetomidine to the background anaesthesia. The results of this study suggest that caution is warranted when using dexmedetomidine as a supplement to propofol-remifentanil TIVA in patients with low blood pressure, since it could result in decreased cerebral oxygenation. Further and larger studies are required for confirmation of these results.

## Additional files

**Additional file 1.** Illustration of experimental flow and data-set of the main experiment. Cerebral perfusion and oxygenation readings, physiological and haemodynamic data, blood gas data, and anaesthesia time at all time points throughout the experiment. PCB, PR-1, PR-2 and PRD are the reported time points used for statistical analysis in the manuscript entitled: *The effect of dexmedetomidine on cerebral perfusion and oxygenation in healthy piglets with normal and lowered blood pressure anaesthetized with propofol*-*remifentanil TIVA*. Analysis of the data collected at the remaining time points will be reported in later manuscript. NIRS: Near infra red spectroscopy; LSCI: Laser speckle contrast imaging; MAP: mean arterial pressure; EtCO2: End-tidal carbon dioxide; FiO2: Fraction of inspired oxygen; (T): data corrected for body temperature; PaCO2: Partial pressure of arterial carbon dioxide; PaO2: Partial pressure of arterial oxygen; HCO3: Hydrogen bicarbonate; Hct: Haematocrit; THbc: Total haemoglobin concentration; NA: data not available.

**Additional file 2.** Sedation protocols. Sedation protocols for intravenous catheter placement on the day prior to the main experiment. One of two intramuscularly injected (im) sedation protocols were used Animal no. 1-11 received protocol 1 (NBP: n = 5, LBP: n = 6), and animal no. 12-16 received protocol 2 (NBP: n = 3, LBP: n = 2).

## Abbreviations

CPO: cerebral perfusion and oxygenation
CRI: constant rate infusion
CSF: cerebrospinal fluid
EtCO_2_: end tidal CO_2_
FiO_2_: fraction of inspired O_2_
im: intramuscular
iv: intravenous
LBP: low blood pressure group
LLCA: lower limit of cerebral autoregulation
LSCI: laser speckle contrast imaging
LSPU: laser speckle perfusion unit
MAP: mean arterial pressure
Max: maximum
Min: minimum
NBP: normal blood pressure group
P_a_CO_2_: partial pressure of arterial CO_2_
P_a_O_2_: partial pressure of arterial O_2_
P_br_O_2_: partial pressure of oxygen in brain tissue
PCB: pre-caval block (time point)
PR: pulse rate
PR-n: propofol/remifentanil TIVA (n: time-point number)
PRD: propofol/remifentanil TIVA + dexmedetomidine CRI (time point)
TIVA: total intravenous anaesthesia
