# Supplementary material for: The effect of dexmedetomidine on cerebral perfusion and oxygenation in healthy piglets with normal and lowered blood pressure anaesthetized with propofol-remifentanil total intravenous anaesthesia
Source: Acta Vet Scand. 2017 May 3;59:27. doi: 10.1186/s13028-017-0293-0 (PMC5415812; doi:10.1186/s13028-017-0293-0)
Supplement: Supplementary file 1 — Additional file 1. Illustration of experimental flow and data-set of the main experiment. Cerebral perfusion and oxygenation readings, physiological and haemodynamic data, blood gas data, and anaesthesia time at all time points throughout the experiment. PCB, PR-1, PR-2 and PRD are the reported time points used for statistical analysis in the manuscript entitled: The effect of dexmedetomidine on cerebral perfusion and oxygenation in healthy piglets with normal and lowered blood pressure anaesthetized with propofol-remifentanil TIVA. Analysis of the data collected at the remaining time points will be reported in later manuscript. NIRS: Near infra red spectroscopy; LSCI: Laser speckle contrast imaging; MAP: mean arterial pressure; EtCO2: End-tidal carbon dioxide; FiO2: Fraction of inspired oxygen; (T): data corrected for body temperature; PaCO2: Partial pressure of arterial carbon dioxide; PaO2: Partial pressure of arterial oxygen; HCO3: Hydrogen bicarbonate; Hct: Haematocrit; THbc: Total haemoglobin concentration; NA: data not available. [file 13028_2017_293_MOESM1_ESM.docx]

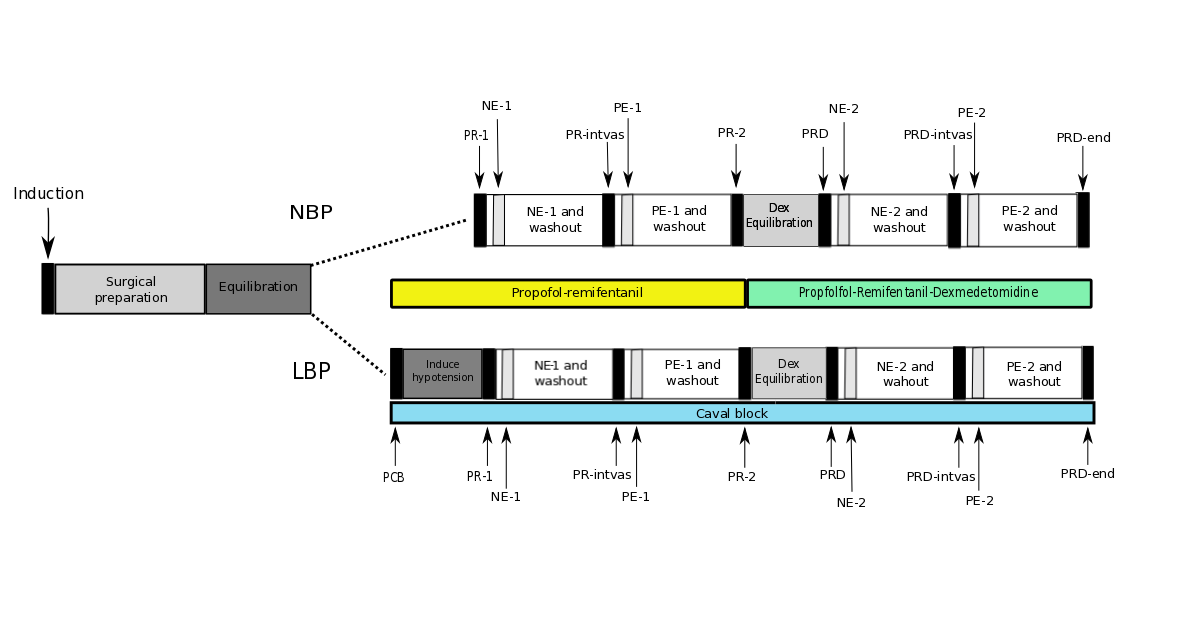

Cerebral perfusion and oxygenation readings, physiological and haemodynamic data, blood gas data, and anaesthesia time at all time points throughout the experiment. PCB, PR-1, PR-2 and PRD are the reported time points used for statistical analysis in the manuscript entitled: *The effect of dexmedetomidine on cerebral perfusion and oxygenation in healthy piglets with normal and lowered blood pressure anaesthetized with propofol-remifentanil TIVA*. Analysis of the data collected at the remaining time points will be reported in later manuscript. PCB: Pre-Caval block; PR-1: baseline during propofol-remifentanil; NE-1: Norepinephrine during propofol-remifentanil; PR-intvas: after norepinephrine and wash-out period during propofol-remifentanil; PE-1: Phenylephrine during propofol-remifentanil; PR-2: after phenylephrine wash-out period/pre-dexmedetomidine during Propofol-remifentanil; PRD: Propofol-remifentanil-dexmedetomidine; NE-2: Norepinephrine during propofol-remifentanil;PRD-intvas: after norepinephrine and wash-out period during propofol-remifentanil-dexmedetomidine; PE-2: Phenylephrine during propofol-remifentanil-dexmedetomidine; PRD-end: after phenylephrine and wash-out period during propofol-remifentanil-dexmedetomidine (end of experiment); NIRS: Near infra red spectroscopy; LSCI: Laser speckle contrast imaging; MAP: mean arterial pressure; EtCO2: End-tidal carbon dioxide; FiO2: Fraction of inspired oxygen; (T): data corrected for body temperature; PaCO2: Partial pressure of arterial carbon dioxide; PaO2: Partial pressure of arterial oxygen; HCO3: Hydrogen bicarbonate; Hct: Haematocrit; THbc: Total haemoglobin concentration.
