# Supplementary material for: The effect of dexmedetomidine on cerebral perfusion and oxygenation in healthy piglets with normal and lowered blood pressure anaesthetized with propofol-remifentanil total intravenous anaesthesia
Source: Acta Vet Scand. 2017 May 3;59:27. doi: 10.1186/s13028-017-0293-0 (PMC5415812; doi:10.1186/s13028-017-0293-0)
Supplement: Supplementary file 2 — Additional file 2. Sedation protocols. Sedation protocols for intravenous catheter placement on the day prior to the main experiment. One of two intramuscularly injected (im) sedation protocols were used Animal no. 1-11 received protocol 1 (NBP: n = 5, LBP: n = 6), and animal no. 12-16 received protocol 2 (NBP: n = 3, LBP: n = 2). [file 13028_2017_293_MOESM2_ESM.docx]

**Sedation protocols:**

1)

- 20 mg/kg ketamine (100 mg/ml Ketaminol® vet., Intervet, Boxmeer, Holland)
- 0.5 mg/kg midazolam (5 mg/ml or Midazolam “B. Braun”, B. Braun Melsungen, Germany).

2)

- 10 mg/kg ketamine (100 mg/ml Ketaminol® vet., Intervet, Boxmeer, Holland)
- 40 µg/kg dexmedetomidine (0.5 mg/ml Dexdomitor®, Orion Pharma Animal Health, Sollentuna, Sweden)
- 0.2 mg/kg butorphanol (10 mg/ml Torbugesic vet., Orion Pharma Animal Health, Sollentuna, Sweden)

Elimination of 98% of all compounds would be expected after 18 hours for both protocols. The reason for using two different protocols were based of the observation that sedation with a combination of ketamine, dexmedetomidine and butorphanol more rapidly established profound sedation with a faster and calmer recovery, than did sedation with a combination of ketamine and midazolam. The change in protocol was therefor of animal welfare grounds.
